# The double task-switching protocol: An investigation into the effects of similarity and conflict on cognitive flexibility in the context of mental fatigue

**DOI:** 10.1371/journal.pone.0279021

**Published:** 2023-02-24

**Authors:** Marcel F. Hinss, Anke M. Brock, Raphaëlle N. Roy

**Affiliations:** 1 ISAE-SUPAERO, Université de Toulouse, Toulouse, France; 2 ENAC, Université de Toulouse, Toulouse, France; University of Cadiz: Universidad de Cadiz, SPAIN

## Abstract

Considerable fundamental studies have focused on the mechanisms governing cognitive flexibility and the associated costs of switching between tasks. Task-switching costs refer to the phenomenon that reaction times and accuracy decrease briefly following the switch from one task to another. However, cognitive flexibility also impacts day-to-day life in many complex work environments where operators have to perform several different tasks. One major difference between typical tasks examined in fundamental studies and real-world applications is that fundamental studies often rely on much more similar tasks, which is not the case for real-world applications. In the latter, operators may switch between vastly dissimilar tasks. Therefore, this behavioural study aims to test if task-switching costs are different for switches between similar and dissimilar tasks. The proposed protocol has participants switch between 2 pairs of two tasks each. Between pairs, there is more dissimilarity, while the two tasks within each pair are more similar. In addition, this study examines the impact of mental fatigue and interference in form of confounding information on cognitive flexibility. To induce mental fatigue the participants’ breaks between blocks will be limited. We expect that dissimilarity between tasks will result in greater task-switching costs.

## 1. Introduction

Cognitive Flexibility, the ability to adapt our behavior and thoughts to the environment, is one of the most important aspects of human cognition [1]. A classical paradigm to study cognitive flexibility is the task-switching paradigm [2–7]. This paradigm requires the participant to switch between two or more relatively simple tasks continuously. Results consistently show that switching between tasks results in task-switching costs (TSC) in the form of increased reaction times and error likelihood following a switch [2–7].

The existence of TSC poses the question of how these effects occur. Two main theories explain this behavioral phenomenon. The Inference view and the Reconfiguration view [8,9]. First proposed by Allport in 1994 the inference view states that during a switch trial the previously activated task set, that is the components relevant to the task (Monsell & Mizon, 2006) [13], interfere with the newly activated task set; The Reconfiguration view [9]on the other hand proposes that task set parameters need to be activated or retrieved from long term memory when a task switch occurs.

In the pursuit of validating one or the other theories, a multitude of variables, such as preparation time, previous interference residual switch costs, working memory, bivalence, similarity and other factors, have been investigated with regard to their relation to TSC [4,10–13]. For a review of the two opposing theories and a summary of the influences of factors on TSC see [11,14].

The similarity factor is of interest for research on cognitive flexibility, especially as most fundamental research has only focused on very similar tasks [7,15]. In these designs, ecological validity is not always given, as task switching in reality may occur between vastly dissimilar tasks. Ecological validity is important with regard to cognitive flexibility, as research has shown that issues with cognitive flexibility have major real-world implications, especially in the domain of Human-Machine Interaction (HMI; [16–20]).

If the fundamental research conducted on cognitive flexibility is to be applied to real-world contexts and problems, the question arises as to what extent these fundamental findings also apply to a realistic context. If this is not the case then it is of interest to see the differences between task-switching in a fundamental context and task-switching in an ecologically valid context [21–23].

This question makes similarity between tasks a critical aspect of understanding cognitive flexibility, as it could be argued that classical task-switching paradigms involve tasks [1] that are more similar than task-switching that is required of operators in complex operations [18].

Some studies have investigated the effects of similarity and closely related effects on task-switching [2,21–24]. In study [2], participants were presented with rectangles of different shapes and colors. Participants had to switch between two sets of two similar tasks and make binary decisions. The tasks were judgements of height, width, hue and intensity. The assumption, which was later validated, was that the width and height, as well as hue and intensity tasks, were more similar.

The responses within that study were univalent (each response possibility, for each task, was mapped onto a different key on the keyboard, or a different vocal response). However, the stimuli were bivalent, meaning that each stimulus could be applied to all tasks. Bivalency is of interest, especially when looking at task-rule incongruent and task-rule congruent responses [11]. In studies where both the stimuli as well as the responses are bivalent (the same responses are used for all keys, and all stimuli can be applied to all tasks), task-rule congruent stimuli (stimuli for which the correct response to the relevant as well as the irrelevant task are the same) are typically faster than task-rule incongruent stimuli (stimuli for which the correct response to the relevant task is not the same as to the irrelevant task [10]. This does not directly apply to the study of [15], as here each response was mapped to a different key and therefore the effect of task-rule incongruency was kept constant. The recent study [21], made use of a similar dimensional design of different tasks. In addition to switching between two tasks, the switch rules could also be different. The work of (Kleinsorge & Heuer, 1999) [23], also uses a dimensional design but here instead of similarity, compatibility of the stimuli is added as a second factor. Further expanding on this is the 2004 study by Kleinsorge [22]. Using a factorial design, participants have to switch between a parity or numerical magnitude task, while either focusing on the numeric value or the number of digits displayed. Participants of this study had to therefore switch between 4 different tasks, however except for switches between tasks where both factors were changed, the tasks all had one factor in common (either the actual task instruction or the part of the stimulus on which to focus).

Several questions arise here: are the incongruency effects that arise in studies using a bivalent design (when the task-relevant cue indicates answer A and the task-irrelevant cue indicates answer B) additive? That is to say, in a task-switching design using 4 different tasks, is there a difference between 1 irrelevant cue biasing towards the incorrect answer or 3 irrelevant cues biasing towards the incorrect answer? What has a stronger impact on TSC, similarity or bivalence/congruency? Does it matter if the biasing cue from a different task comes from a more similar or a more dissimilar task?

Beyond exploring these questions that have a very fundamental ft in understanding cognitive flexibility, there is also an interest when going back to more ecological concerns. Task-Switching in an ecological context may involve switching between tasks where similarity and bivalence are independent of each other. The here proposed study is also lacking in ecological validity. However, investigating the mechanisms of cognitive flexibility in the context of more or less dissimilar tasks, may facilitate future research to experiment with vastly dissimilar tasks that better resemble the real world.

With the goal of paying attention to factors vital to ecological validity mental fatigue and time on task (TOT) were identified as important covariates. While studies with a multitude of different tasks often show an increase in reaction time and a decrease in accuracy as TOT [25–27], studies on cognitive flexibility, show that the increase in TSC is significantly bigger, than the overall increase in reaction time and decrease in accuracy [28–31]. Furthermore, mental fatigue has been identified as a risk factor in several types of complex operations, that simultaneously requires the operator to maintain a degree of cognitive flexibility [20,32–34] Beyond complex operations, deficits in cognitive flexibility are also associated with several psychiatric/ psychological conditions. Research has shown deficits in cognitive flexibility associated with Parkinson’s disease, Schizophrenia, attention deficit/Hyperactivity Disorder (ADHD) and Depression [35,36] The results of this study may also help understand the impact the pathologies have on the lives of those affected, in the context of cognitive flexibility.

NOTE: For the remainder of this preregister we will no longer use the term bivalency to explain, but rather the terms conflict and congruency. This is because in the experimental design 4 different levels of conflict are planned. These consist of a level where only the task-relevant cue is present and three conditions were also other task-irrelevant cues are present. However, it was decided that the task’s irrelevant cues are always incongruent. This decision was made to allow the evaluation of accuracy in addition to reaction time as a dependent variable. If we were to involve trials where a task-irrelevant cue indicates the same correct answer as the task-relevant cue, it would be impossible to know if the participant, given that he responds correctly, if this correct response was also based on the correct cue or the task-irrelevant cue.

## 2. Hypotheses (required)

2.1. Task switching cost
  2.1.1. Following a switch of the task, the reaction time on said trial is higher than the average reaction time on trials of the same task where previously no switch occurred.
  2.1.2. Following a switch of the task, accuracy on said trial is lower than the average accuracy on trials of the same task where previously no switch occurred.
2.2. Similarity Effects: Task Switching costs (hypothesis 1; Reaction time and accuracy), are more pronounced if the switch is between two more dissimilar tasks.
2.3. Conflict Effects:
  2.3.1. An incongruent stimulus will result in a higher reaction time regardless of whether the trial is a switch trial or a repetition trial.
  2.3.2. An incongruent stimulus will result in a lower accuracy regardless of whether the trial is a switch trial or a repetition trial.
2.4. Mental Fatigue:
  2.4.1. As time on task increases, the reaction times will increase regardless of whether the trials are switch trials or repetition trials.
  2.4.2. As time on task increases, error likelihood will increase regardless of whether the trials are switch trials or repetition trials.
  2.4.3. As time on task increases, Task Switching costs (hypothesis 1) will increase

Design Plan

## 3. Study type: Experiment

    

## 4. Ethics statement

Ethical Consent was obtained from the Comité d’Éthique de la Recherche—CER at the Université Fédérale Toulouse Midi-Pyrénées Approval for written consent was obtained on June 2. 2020 (ID 2022–521).

## 5. Blinding

Within subjects design. Every participant will complete the same trials as all the other participants.

Participants will be aware, that the goal of the experiment is to study cognitive flexibility and that mental fatigue plays a role. Participants will not be made aware of the hypotheses to avoid demand characteristics.

Personnel interacting with the data and the participants are aware of the goal as well as the experimental design of the study (necessary for the data analysis).

## 6. Is there any additional blinding in this study?

No.

## 7. Study design

https://www.protocols.io/view/dts-tot-protocol-n92ldzn88v5b/v1

The DTS protocol consists of 4 subtasks, 2 based on the numeric value of the displayed stimuli and two based on the visual appearance of the stimuli. Within the numeric subtasks, participants are either performing the LOW/HIGH task or the EVEN/ODD task.

For the LOW/HIGH task participants have to judge, whether a single number is higher or lower than 5 and indicate their response with a keystroke. In the EVEN/ODD task participants have to decide if a stimulus (again a single number, but located spatially different) is even or odd. Again, participants are instructed to respond with a keystroke.

**Fig 1:**
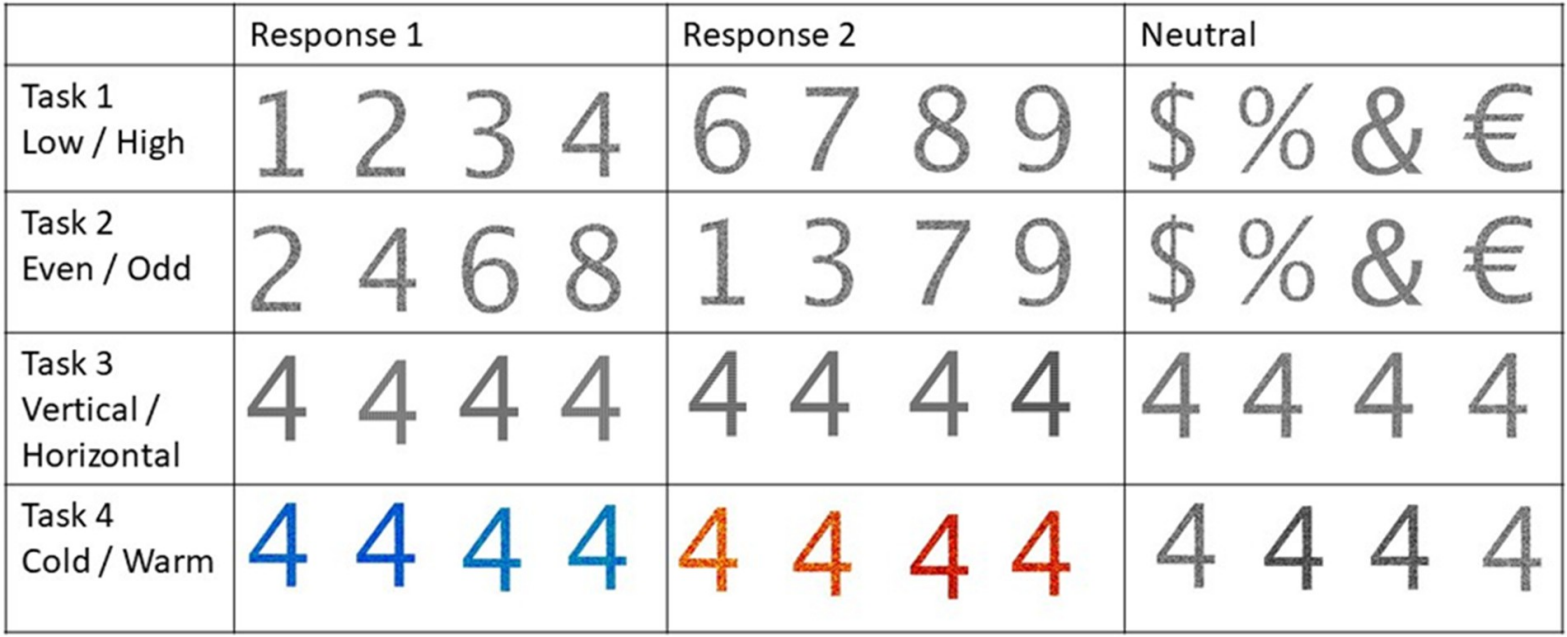
Examples of different stimuli for all subtasks of the DTS protocol.

**Fig 2:**
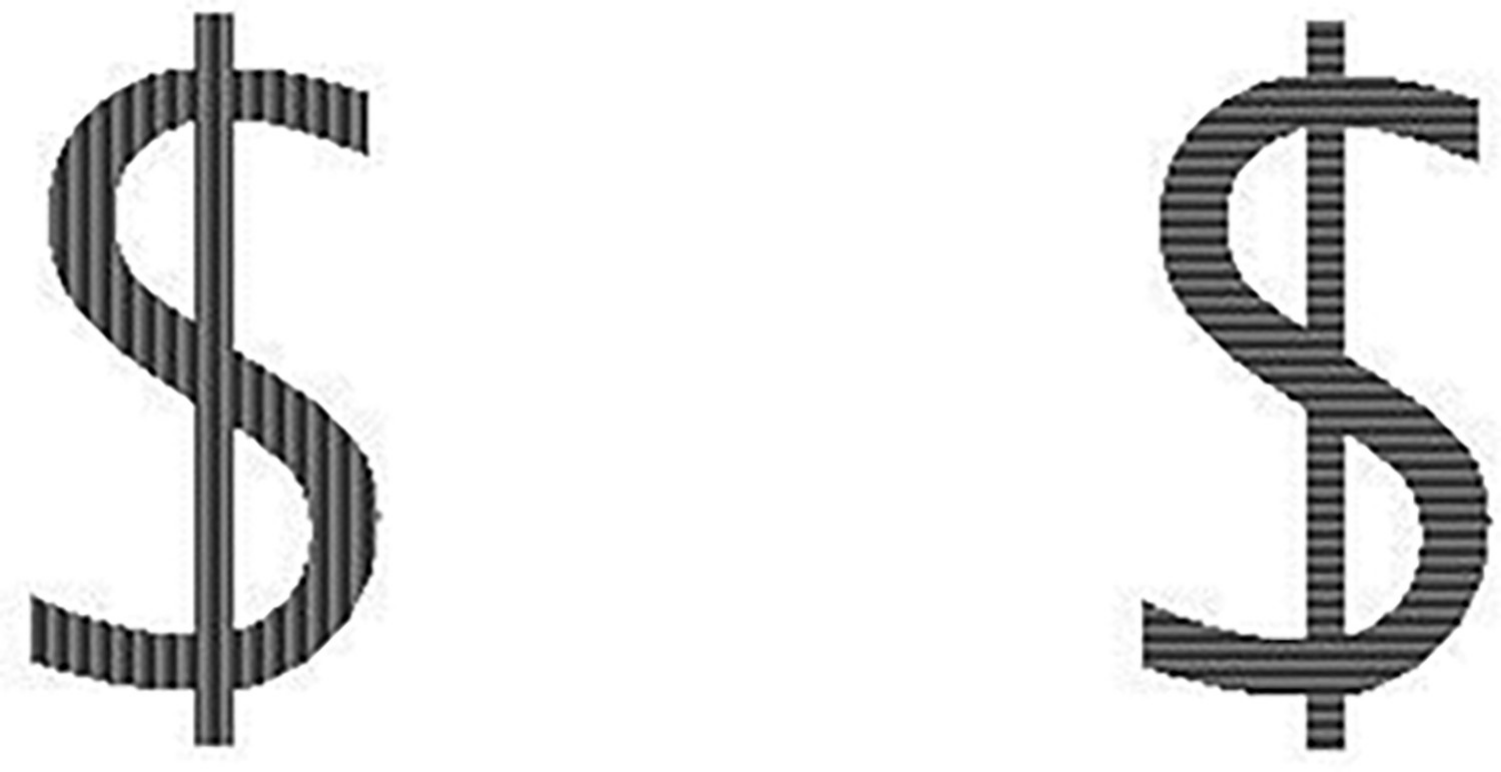
Larger example of stimuli with cue relevant for Task 3.

For the visual tasks; participants have to either perform the COLD/HOT task, where they have to decide whether the stimulus is presented in either a hot or a cold color, or they have to perform the VERTICAL/HORIZONTAL task, in which the orientation of a pattern has to be determined (see Figs 1 and 2 for an example array of stimuli for each condition, instructions can also be displayed in French).

During the task, participants have to perform consecutive trials, where the task and stimuli will change quasi-independently from trial to trial.

For each trial, participants are presented with 3 quasi-independent pieces of information. They are presented with an instruction for the current trial in form of the task name (eg. VERTICAL/HORIZONTAL, presented 300ms before the actual stimulus) and two stimuli in form of signs or numbers. Based on the stimuli and the instruction participants are instructed to then respond as fast as possible, using either the S or the L key on a keyboard (keys were selected as they are located at a comfortable distance on the keyboard, in order to avoid straining the hands of the participants, keys will be marked with stickers for participant ease).

In total there are 96 different conditions for each trial (4 Tasks * 3 Switching conditions * 4 Congruency Conditions * 2 Response types). However, as differences in performance of individual tasks, as well as response type, are not hypothesized to have any impact on the performance of participants, 12 main conditions remain.

*Task-Switching Conditions*:

Each trial (n) is classified into one of the three Task-Switching Conditions depending on the task in trial n-1. If the task remains the same, the trial is a repeat trial. If the task switches, but remains within the same domain (LOW/HIGH to EVEN/ODD or VERTICAL/HORIZONTAL to COLD/HOT and vice versa), it is considered an internal switch trial. Finally, switches from one domain into the other (LOW/HIGH or EVEN/ODD to either VERTICAL/HORIZONTAL or COLD/HOT and vice versa) are considered external switch trials.

Note that patterns in Task 3 are not clearly visible in the document, please refer to Fig 2.

*Congruency Conditions*:

Depending on the stimuli that are presented a trial may evoke different degrees of conflict as to the correct response required. This occurs when the **task-relevant information of the stimulus** indicates, that eg. response 1 is correct, while in the presence of a non-relevant cue biasing the response in the incorrect condition. Here 4 different conditions exist.

**Fig 3:**
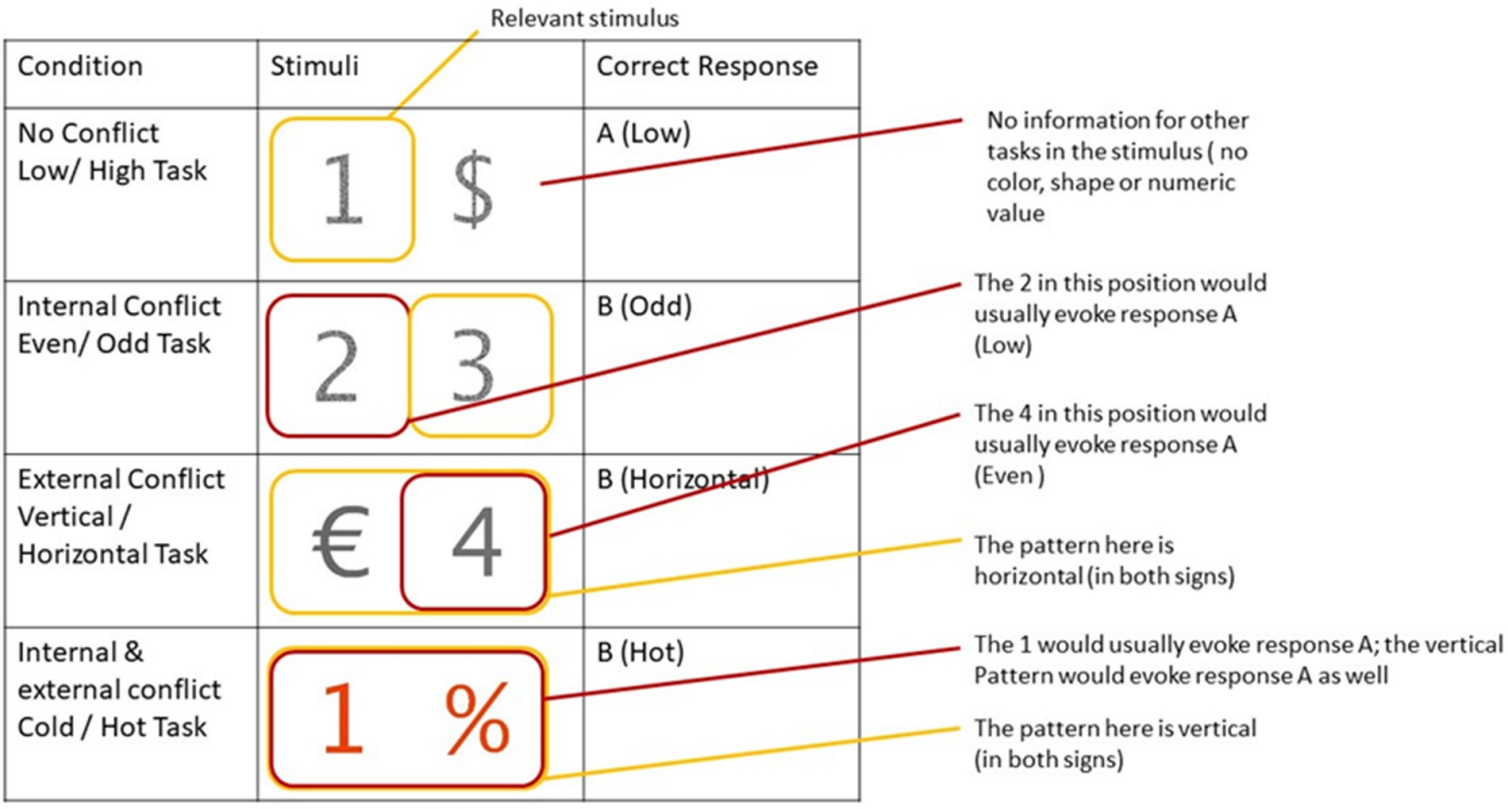
Examples of the different congruency conditions.

In the no-conflict condition, a stimulus only presents one piece of information, the relevant one. In Fig 3 the stimuli in the no-conflict condition provide no information relevant to each of the remaining 3 tasks.

The internal conflict condition adds a piece of information that would indicate the opposite response in the other task of the same domain. In the example, the 3 is the relevant number, due to its position, as the participant is indicated to perform the Even/ Odd task (which is always based on the second number), but the other stimulus (, the 2) would require the participant to respond with the other response if the task were the Low/High task (the task for which, the first number is relevant).

The external conflict condition is similar, with the only difference being, that the non-relevant task is not from the same domain, but is one (and always only one) of the tasks from the other domain.

The final condition, internal & external conflict combines the two other conflict conditions, where both the other internal task and one (and always only one) of the external task, bias the participant towards an incorrect response.

In order to avoid participants using task-irrelevant cues as a strategy to increase performance (Logan & Zbrodoff, 1979) half of the trials will include congruent task-irrelevant stimuli and half of the trials will include task-irrelevant incongruent stimuli. Task irrelevant stimuli will always all be either congruent or incongruent. The downside of this is, that it becomes impossible, during trials with congruent task-irrelevant stimuli, to determine whether a correct or incorrect answer occurred due to the task-relevant or task-irrelevant stimulus. For any analysis that requires this, only the incongruent task-relevant trials may be used.

The locations of the relevant stimuli for the numeric tasks were chosen to be Low/High on the left and Even/Odd on the right for the following reason. Should participants perceive two single digits not as e.g. seven and one but as seventy-one, this would not change the responses. As the Even/Odd relevant number is located (right, or for this discussion 2.) it determines whether a number is odd or even, independent from the number to the left of it (that is to say, both 1 and 71 are odd numbers). In a similar way, the size of the first number is somewhat independent of the second number (7 is larger than 5, but 71 is also larger than any number in the fifties).

**Fig 4:**
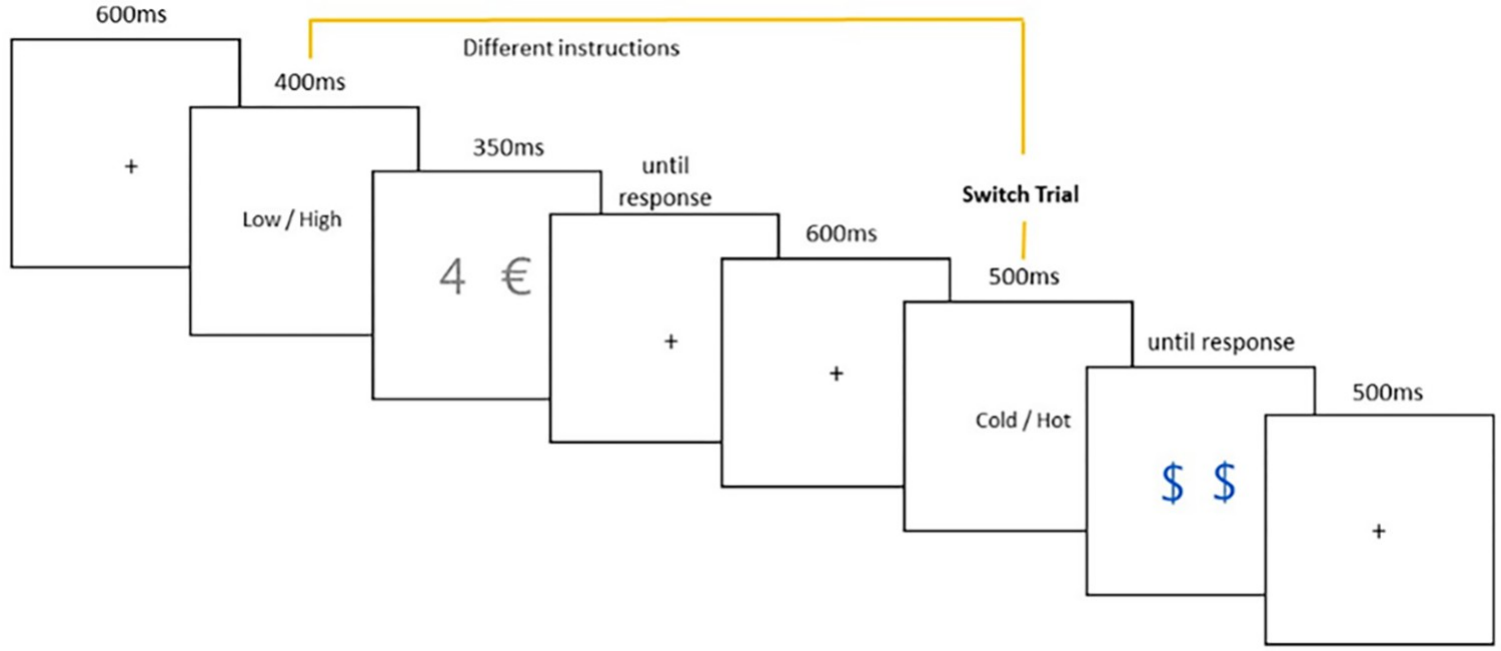
Example of a single trial.

Trial Setup:

Each trial will begin with a fixation cross in the middle of the screen for 350-850ms (600ms with a 250ms jitter), followed by trial instruction (400ms). Finally, participants are presented with the stimulus (350ms), where they are able to respond with a keystroke. If participants take longer than 350ms to respond the stimulus disappears. Once a response is recorded, a new trial begins (See Fig 4).

The task was computed using PsychToolBox-3 (http://psychtoolbox.org/) in Matlab and will be presented on a desktop computer.

## 8. Randomization

Participants are not randomized (within-subjects design), however, the trials that participants will complete are quasi-random. That is to say, trials will appear in a random order, which ensures a balanced design and equal probabilities of each trial occurring.

Sampling Plan

## 9. Existing data

Registration prior to the creation of data. Ethical Approval was obtained on June 2. 2022 (projet 2022–521 Université Fédérale Toulouse Midi-Pyrénées)

## 10. Data collection procedures

Recruitment:

*Recruitment method*: the participants are recruited by e-mail and via paper announcements in the institute buildings.

*Place of recruitment*: participants will be recruited at ISAE-SUPAERO

*Selection criteria*: Subjects must be between 18 and 30 years old, have a level of study baccalaureate minimum, have a normal or corrected-to-normal vision and hearing, be affiliated with social security and have signed an informed consent form.

*Exclusion criteria*: Protected persons, a person outside the ISAE, presence of known neuropsychological disorder, significant visual or hearing impairment, taking psychotropic medication or substance, g-test positive, nursing woman. In addition, due to the nature of the experiment, colorblind people will be excluded from participation.

As recommended to minimize the risk of an invasion of privacy, we will list all the criteria (inclusion and/or exclusion) and ask participants whether they meet all the criteria, rather than asking them whether they meet each criterion one by one. Due to the nature of the experiment, participants will also complete a test for colorblindness prior to signing the inclusion/exclusion criteria. To do so we will use the Ishihara test for colorblindness (Hardy et al., 1945), which is widely applied and validated within science and medicine.

Compensation for subjects:

Participants will be paid in gift vouchers (Illicado, illicado.com). Upon completion participants will receive a voucher worth 20€ (5€ for the first training session, and 15€ (for the longer testing session). Should participants drop out after the first session they will be informed that they have the right to receive the 5€ voucher nevertheless.

**Protocol:** In the experiment participants will complete the DTS protocol as presented above.

After the arrival of the participant for the first session, the participant will be asked to complete the Ishihara colorblindness test, before filling in the informed consent and information sheets. The participant will be informed, that they are permitted to ask questions at any time, and are also asked in what language they would like to perform the task (French or English). They will then fill in the Demographics questionnaire, the Edinburgh Handedness questionnaire and a KSS as well as the SPS scale. Next, the participant will be familiarized with the DTS protocol, and perform a training session, until the participants have acquired an adequate level of skill in the task.

For the training session, participants will perform at least 5 blocks (96 trials each, 3,5 min without feedback, 4 minutes with feedback) of the DTS protocol. During the first block, participants will receive feedback following each trial, to increase their individual learning rates. Subsequent blocks will exclude the feedback to completely mimic the experimental conditions. Additional blocks of training may be added following the 5 blocks to ensure adequate task performance. Additional training may be added, if the last completed block of trials, still shows a significant improvement over the preceding trial. Additional blocks will only be added with the explicit consent of the participant.

After the training session, the participant will again fill in a KSS as well as an SPS scale.

The second session will be scheduled no longer than 14 days after the first session. The procedure of the second session is largely similar to that of the first session. Participants will perform a much shorter second training session intended to help the participant remember the task. Participants will complete half a block of trials (48 trials) with feedback and another half block without feedback. Next participants will fill in a KSS and SPS scale and perform the experimental DTS protocol as detailed above. Participants will complete 5 Blocks of 336 trials each. In between blocks participants will have a short break of 28 seconds as proposed by Lim et al., 2016, to avoid recovery from the TOT effects.

**Fig 5:**
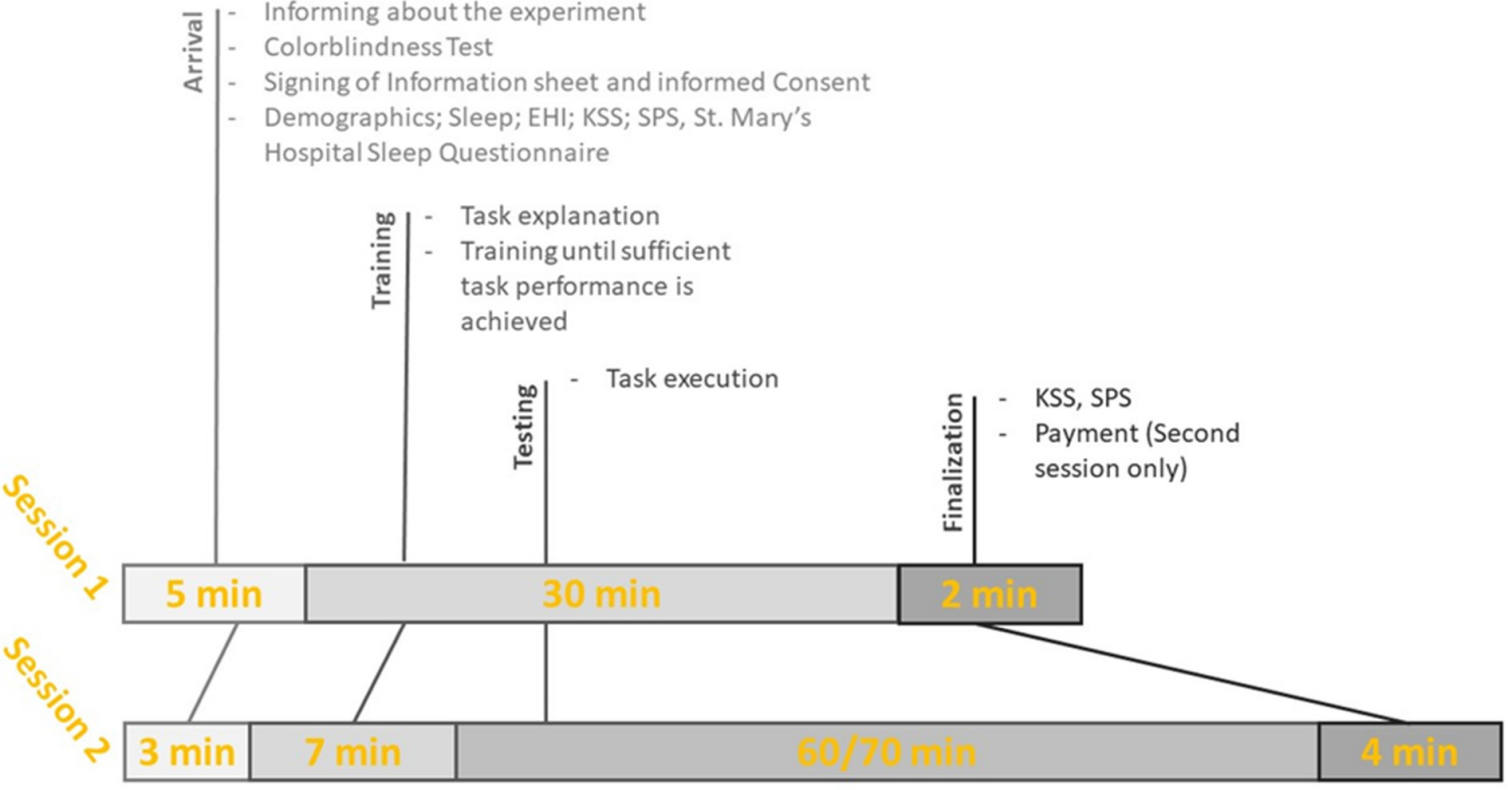
Timeline of the two sessions for Experiments 1 and 2.

Upon completion of the task participants will again fill in the KSS and SPS scale, before being thanked for their participation and paid (See Fig 5).

## 11. Sample size (required)

The approximate range of required participants was determined using G*Power [37] Based on the reported statistics [3,15]. Their reported effect sizes ranged from 0.84 to 1.81 (f-squared). For a one-tailed test with an alpha of 0.05. The sample size required ranges from 15–25 participants. These numbers are only approximations, as the effects that we intend to observe are not exactly the same as the ones reported in the aforementioned studies. We intend to collect data from 30 participants, in order to assure an adequate effect size.

Variables

## 12. Manipulated variables (optional)

Stimuli:

**Numeric Value:** The value of the stimuli that are presented is manipulated. Participants with see combinations of the numbers 1,2,3,4,6,7,8 and 9 and symbols %, &, € and $.

**Color:** The stimuli color is manipulated to either be hot (red/orange hues), cold (blue/ green hues) or neutral (grey hues).

**Pattern:** The pattern of the stimuli is manipulated and can either show a horizontally oriented pattern, a vertically oriented pattern or a random pattern.

**Task:** The task that participants have to complete given the stimuli can be adapted as can be seen in Figs 1 and 2 the four tasks are Low/High, Even/Odd, Cold/Hot, Vertical/Horizontal.

**Conflict**: Depending on what stimuli are presented together the experiment manipulates the degree of conflict. See Fig 3.

**Conflict Congruency:** The conflict stimuli can be either congruent or incongruent with the task relevant stimulus.

Covariate:

Time on task (TOT), how long the participant has been performing the task, will be incorporated into the analysis as a key covariate indicator of mental fatigue.

## 13. Measured variables (required)

**Accuracy:** The number of correct answers during the task will be recorded.

**Reaction Time:** The reaction time on each trial will be recorded. Reaction time here is measured as the time from stimulus presentation to response (keystroke).

**Subjective Questionnaire Data:** The data from several subjective questionnaires will be recorded.

Note: All questionnaires will be presented to the participants in a paperless digitalized form.

Demographics Questionnaire:

The demographics questionnaire will include questions assessing age, gender and occupation. This questionnaire can be found in Appendix 5 in S1 Appendix.

Edinburgh Handedness Inventory:

The shortened version of the Edinburgh Handedness Inventory encompasses 4 items (e.g. writing) scored on a 5-point scale ranging from:” Always right; Usually right; Both equally; Usually left; Always left”. This shortened version has been shown to be a faster measure while maintaining its reliability [38]. A copy of the inventory is attached as Appendix 8 in S1 Appendix.

### KSS

The KSS was developed to measure subjective sleepiness at any given time. It is a simple 9-point scale ranging from “extremely 1 = alert” to “9 = extremely sleepy–fighting sleep”. The scale has been validated and used frequently and provides a simple and fast measure that at the same time provides a reliable result [39]. The scale is attached in Appendix 7 in S1 Appendix.

SPS:

SPF: The estimation of fatigue will be performed using the Samn-Perelli Fatigue scale (SPF; [40]). This scale consists of 7 points and is used in aviation to assess crew fatigue. The French version comes from a report by the International Civil Aviation Organization [41]), but has not been psychometrically validated (see Appendix 6 in S1 Appendix).

Ishihara Colorblindness Test:

The Ishihara test for colorblindness [41] encompasses stimuli, that can determine the degree as well as the type of colorblindness, a person has. It will be presented in printed form, using a validated version of the test made available by the US department of infectious diseases (see Appendix 9 in S1 Appendix). Participants will be informed, that for a medically accurate test, a licensed physician is required. Should the suspicion of colorblindness arise during the assessment participants will be informed and advised to seek out a medical professional.

**The St Mary’s Hospital Sleep Questionnaire** (QSN; [42]) will be used to estimate the quality of sleep on the previous night. A translated version of this questionnaire has been produced (see Appendix 10 in S1 Appendix). The scoring of this questionnaire is not standardized due to the use of Likert scales and free responses. In addition, two questions regarding caffeine consumption have been added to this questionnaire.

Analysis Plan

The Analysis will be performed using Mixed (Multi-Level) Regression, as this allows for accounting for the effects of participants as well as tasks.

A first mixed multilevel regression will be performed on the dependent variable of RT (in MS).

The **independent Variables** are Trialtype (3 Levels), Conflict (4 Levels), Conflict Congruency (Binary) and Response (Binary)

The **dependent variable** is RT (in MS).

Time on Task (Time since task onset) will be included as a **covariate**

In a second Mixed multilevel logistic regression accuracy will be analyzed.

The **independent Variables** are Trialtype (3 Levels) and Conflict (4 Levels) and Conflict Congruency (Binary)

The **dependent variable** is Accuracy (Binary)

Time on Task (Time since task onset, and or trial number) will be included as a **covariate**

In order to account for both participants as well as task effects, both will be dummy coded and included as IV effects in the Mixed Regression for both analyses. In order to best determine the most fitting covariance structure, the analysis will first be performed with a random intercept + ARMA (or AR1). Random slopes may be added if the fixed model is reduced or following a test for slope variance of the IV effects.

Expected Results: The expected results based on the hypotheses are detailed in Table 1.

**Table 1:** Expected results with corresponding hypotheses.

	Hypothesis	Expected effect
Task-Switching Costs	Following a switch of the task, reaction time on said trial is higher than the average reaction time on trials of the same task where previously no switch occurred	The effect of trial-type (switch vs repeat) is significant in the Mixed Multilevel Regression (higher RT for switches)
	Following a switch of the task, accuracy on said trial is lower than the average accuracy on trials of the same task where previously no switch occurred.	The effect of trial-type (switch vs repeat) is significant in the Mixed Multilevel Logistic Regression (lower accuracy for switches)
Similarity effects	For all switch trials, reaction time will be longer if the switch occurs between dissimilar tasks	The interaction effect between trial-type (switch vs repeat) and similarity is significant in the Mixed Multilevel Regression (higher RT for dissimilar switches)
	For all switch trials, accuracy will be lower if the switch occurs between dissimilar tasks	The interaction effect between trial-type (switch vs repeat) and similarity is significant in the Mixed Multilevel Logistic Regression (lower accuracy for dissimilar switches)
Conflict effects	An incongruent stimulus will result in a higher reaction time regardless of whether the trial is a switch trial or a repetition trial.	The effect of congruency (congruent vs incongruent) is significant in the Mixed Multilevel Regression (higher RT for incongruent trials)
	An incongruent stimulus will result in a lower accuracy regardless of whether the trial is a switch trial or a repetition trial.	The effect of trial-type (congruent vs incongruent) is significant in the Mixed Multilevel Logistic Regression (lower accuracy for incongruent trials)
Mental fatigue	As time on task increases, the reaction times will increase regardless of whether the trials are switch trials or repetition trials.	The covariate of TOT is significant in the Mixed Multilevel Regression (higher RT over time)
	As time on task increases, error likelihood will increase regardless of whether the trials are switch trials or repetition trials.	The covariate of TOT is significant in the Mixed Multilevel Logistic Regression (lower accuracy over time)
	Following a switch of the task, reaction time on said trial is higher than the average reaction time on trials of the same task where previously no switch occurred	The interaction effect between trial-type (switch vs repeat) and TOT is significant in the Mixed Multilevel Regression (higher RT for switches as TOT increases)
	Following a switch of the task, accuracy on said trial is lower than the average accuracy on trials of the same task where previously no switch occurred.	The interaction effect between trial-type (switch vs repeat) and TOT is significant in the Mixed Multilevel Logistic Regression (lower accuracy for switches as TOT increases)

## 14. Inference criteria (optional)

The general inference criterion is a p-value of p < .05. In Multiple comparisons, we will adjust that criterion based on the adjusted Bonferroni criterion for multiple comparisons.

## 15. Data exclusion (optional)

To detect and reject outliers two steps will be implied. First trials with reaction times of above 5 seconds will be rejected and counted as misses. In addition, an outlier detection will be performed based on the interquartile range criterion. This will be done for trials grouped by condition.

## 16. Missing data (optional)

Trials with missing or incomplete data will be removed. However, due to the design, it is not expected to occur. Furthermore, due to a large amount of trials single missing trials should not affect the statistical validity of the conclusions.

## 17. Exploratory analysis (optional)

We expect that responses on subjective questionnaires as well as demographics may be valuable covariates partially explaining the variations in reaction time and accuracy, within an exploratory analysis we, therefore, aim to investigate such possible links.

A frequently reported effect during behavioral tasks is the so-called rebound effect, of a lower performance following an error. This may also be analyzed in the context of explaining task-switching costs.

## 18. Other

    

## Supporting information

S1 Appendix (DOCX)
