# Supplementary material for: The double task-switching protocol: An investigation into the effects of similarity and conflict on cognitive flexibility in the context of mental fatigue
Source: PLoS One. 2023 Feb 24;18(2):e0279021. doi: 10.1371/journal.pone.0279021 (PMC9955658; doi:10.1371/journal.pone.0279021)
Supplement: S1 Appendix — (DOCX) [file pone.0279021.s001.docx]

**APPENDIX 1 - INFORMATION SHEET AND INFORMED CONSENT**

**Project title:** DTS-TOT

**Tenured researcher, scientific coordinator of the project:**

Raphaëlle N. Roy

raphaelle.roy@isae-supaero.fr

+33 (0)5 61 33 87 20

Associate Professor (tenured) of Neuroergonomics & Physiological Computing

Department of Conception and Control of Aeronautical and Spatial Vehicles

ISAE-SUPAERO

**Place of research:** ISAE-SUPAERO, 10 avenue Edouard Belin 31400 Toulouse

**Goal of the research project:**

The experiment is aimed at investigating the effects of similarity between tasks on the capability of an individual to switch between different tasks within the context of mental fatigue.

**What we expect of you (methodology):**

If you agree to participate in this study, you will be invited to come to our laboratory for two sessions. First, you will be asked to fill in questionnaires assessing demographics, handedness, fatigue and colourblindness. Following this part, you will be familiarized with the Double Task-Switching Protocol (DTS). This task involves 4 different subtasks, that require you to discriminate stimuli based on their numeric value and their appearance. The stimuli will be presented on a computer. You will have time to familiarize yourself with the task. This is all done during the first session. In the second session (which takes part between 1-2 weeks after the first session) you will once again be asked to fill in a few questionnaires before completing another smaller training session. Once your training is finished you will perform the whole task. There are only very brief breaks during the task. After completion of the task, you may have to fill in several more questionnaires assessing your current mental state after task completion.

The first session will take less than an hour (one-hour maximum, should you require extra training), while the second session takes around an hour and a  half.

**Your right to withdraw from the research programme at any time:**

If you agree to participate in this study you must understand that your participation is voluntary and that you can withdraw or stop participating at any time. Any decision to withdraw will have no impact on your academic career at ISAE-SUPAERO or any other university. Furthermore, the withdrawal will have no impact on any future relations associated with ISAE-SUPAERO or the DCAS department.

**Your right to confidentiality and respect for privacy:**

*1. The data obtained will be processed in the utmost confidentiality;*

*2. Your identity will be concealed using a randomly assigned number;*

*3. No other information will be disclosed which could reveal your identity;*

*4. All the data will be kept in a secured server and paper documents in a secured place. Only the scientific supervisor and associate researchers will be allowed access;*

*5. Note that the rigorous anonymity process here applied makes it impossible to correct or destroy your data after anonymization*

*In addition, and if you consent, your anonymized data will be made available to other researchers for research purposes, while ensuring the necessary confidentiality and protection of your data.*

**Benefits:**

This study will allow us to better understand human cognition and cognitive flexibility in situations of mental fatigue. This is of major importance as flexibility is one of the characteristics that are best associated with intelligent behaviour, and understanding more aspects will allow us to design systems, that preserve this flexibility in face of adverse factors such as fatigue.

**Possible risks:**

*To the best of our knowledge, this research does not involve any risk or discomfort other than those involved in everyday life.*

**Publication:**

*This research will be shared during conferences and published in conference reports and articles in academic journals.*

**Your right to ask questions at any time:**

*You can ask questions about the research at any time by contacting the project’s scientific coordinator via email at:*

marcel.hinss@isae-supaero.fr

**Consent before participation:**

By signing the consent form, you certify that you have read and understood the information above, that we have provided satisfactory answers to your questions and duly informed you that you are free to withdraw your consent or withdraw from the research at any time, without prejudice.

**To be filled in by the participant:**

I have read and understood the information above and I freely agree to participate in this research study.

Date: ………………………………………………

 
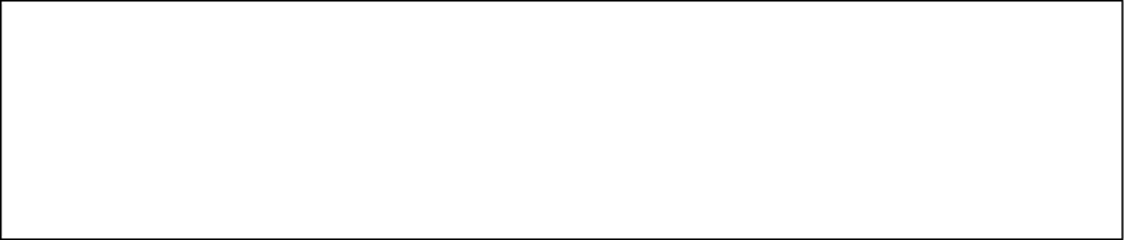


Participants' Name and signature:  …………………………………………………………………….

Experimenter’s name and signature: …………………………………………………………………

You will be given a copy of this document; another copy will be kept in the file.

**APPENDIX 2 - INFORMED CONSENT FORM FRENCH**

Titre du projet : DTS-TOT

Chercheur titulaire, coordinateur scientifique du projet :

Raphaëlle N. Roy

raphaelle.roy@isae-supaero.fr

+33 (0)5 61 33 87 20

Professeur associé (titulaire) de neuroergonomie et d'informatique physiologique

Département Conception et Contrôle des Véhicules Aéronautiques et Spatiaux

ISAE-SUPAERO

**Lieu de recherche** : ISAE-SUPAERO, 10 avenue Edouard Belin 31400 Toulouse

**Objectif du projet de recherche :**

L'expérience vise à étudier les effets de la similarité entre les tâches sur la capacité d'un individu à passer d'une tâche à l'autre dans un contexte de fatigue mentale.

**Ce que nous attendons de vous (méthodologie) :**

Si vous acceptez de participer à cette étude, vous serez invité à venir dans notre laboratoire pour deux séances. Tout d'abord, il vous sera demandé de remplir des questionnaires évaluant les données démographiques, le sens de la main, la fatigue et le daltonisme. Ensuite, vous vous familiariserez avec le protocole de changement de tâche double (DTS). Cette tâche comporte 4 sous-tâches différentes, qui vous demandent de discriminer des stimuli en fonction de leur valeur numérique et de leur apparence. Les stimuli seront présentés sur un ordinateur. Vous aurez le temps de vous familiariser avec la tâche. Tout cela se fait au cours de la première session. Lors de la deuxième session (qui se déroule entre 1 et 2 semaines après la première), vous devrez à nouveau remplir quelques questionnaires avant de suivre une autre petite session d'entraînement. Une fois la formation terminée, vous effectuerez l'ensemble de la tâche. Il y a peu de pauses pendant la tâche. Une fois la tâche terminée, vous devrez peut-être remplir plusieurs autres questionnaires pour évaluer votre état mental actuel après la tâche. La première session dure moins d'une heure (une heure maximum, si vous avez besoin d'une formation supplémentaire), tandis que la seconde dure environ une heure et demie.

**Votre droit de vous retirer du programme de recherche à tout moment :**

Si vous acceptez de participer à cette étude, il est essentiel que vous compreniez que votre participation est volontaire, et que vous pouvez vous retirer ou arrêter de participer à tout moment. Toute décision de retrait n'aura aucun impact sur votre carrière académique à l'ISAE-SUPAERO ou dans toute autre université. De plus, le retrait n'aura aucun impact sur toute relation future associée à ISAE-SUPAERO ou au département DCAS.

**Votre droit à la confidentialité et au respect de la vie privée :**

1. Les données obtenues seront traitées dans la plus grande confidentialité ;

2. Votre identité sera dissimulée au moyen d'un numéro attribué de manière aléatoire ;

3. Aucune autre information susceptible de révéler votre identité ne sera divulguée ;

4. Toutes les données seront conservées dans un serveur sécurisé et les documents papier dans un endroit sécurisé. Seuls le superviseur scientifique et les chercheurs associés seront autorisés à y accéder ;

5. Notez que le processus rigoureux d'anonymat ici appliqué rend impossible la correction ou la destruction de vos données après anonymisation.

En outre, et si vous y consentez, vos données anonymisées seront mises à la disposition d'autres chercheurs à des fins de recherche, tout en assurant la confidentialité et la protection nécessaires de vos données personnelles.

**Avantages :**

Cette étude nous permettra de mieux comprendre la cognition humaine et la flexibilité cognitive dans des situations de fatigue mentale. Ceci est d'une importance majeure car la flexibilité est l'une des caractéristiques les mieux associées au comportement intelligent, et la compréhension de plus d'aspects nous permettra de concevoir des systèmes qui prévoient cette flexibilité face à des facteurs défavorables comme la fatigue.

**Risques possibles :**

A notre connaissance, cette recherche ne comporte aucun risque ou inconfort autre que ceux liés à la vie quotidienne.

**Publication :**

Cette recherche sera partagée lors de conférences et publiée dans des rapports de conférences et des articles dans des revues académiques.

**Votre droit de poser des questions à tout moment :**

Vous pouvez poser des questions sur la recherche à tout moment en contactant le coordinateur scientifique du projet par e-mail à l'adresse suivante :

*marcel.hinss@isae-supaero.fr*

**Consentement préalable à la participation :**

En signant le formulaire de consentement, vous certifiez que vous avez lu et compris les informations ci-dessus, que nous avons apporté des réponses satisfaisantes à vos questions et que vous avez été dûment informé que vous êtes libre de retirer votre consentement ou de vous retirer de la recherche à tout moment, sans préjudice.

**A remplir par le participant :**

J'ai lu et compris les informations ci-dessus et j'accepte librement de participer à cette étude de recherche.
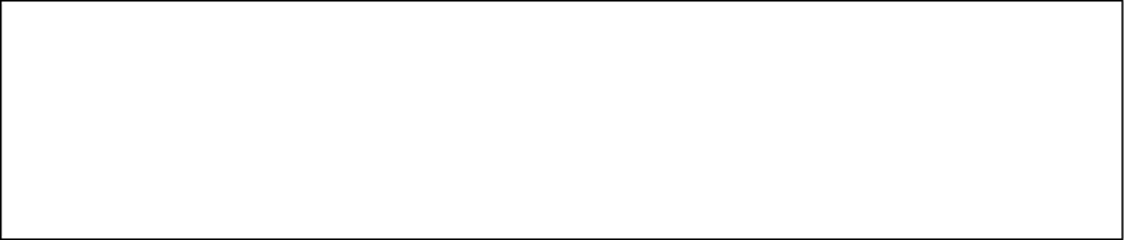


**Date :** ......................................................

**Nom et signature du participant :** ....................................................................................

**Nom et signature de l’expérimentateur :** .........................................................................

Une copie de ce document vous sera remise ; une autre copie sera conservée dans le dossier.

**APPENDIX 3 - Inclusion Criteria  English**In order to minimize privacy infringement, we are listing all the criteria (inclusion and/or exclusion) and asking if you meet all of them at once.

Inclusion Criteria: Please answer YES or NO if you meet or not all the listed points:

·         Age between 18 and 60 years old,

·         you have a level of study baccalaureate minimum,

·         you have normal (or corrected) vision and hearing,

·         you are affiliated with social security,

·         you have signed an informed consent form.

Answer: ……………..

Exclusion Criteria: Please answer YES or NO if you meet or not all the listed points:

·         protected person;

·         presence of known neuropsychological disorder;

·         significant visual or hearing impairment;

·         taking psychotropic medication or substance;

·         epilepsy;

- colorblindness

·         pregnancy;

·         nursing;

·         frequent headaches;

Answer: ……………..

 
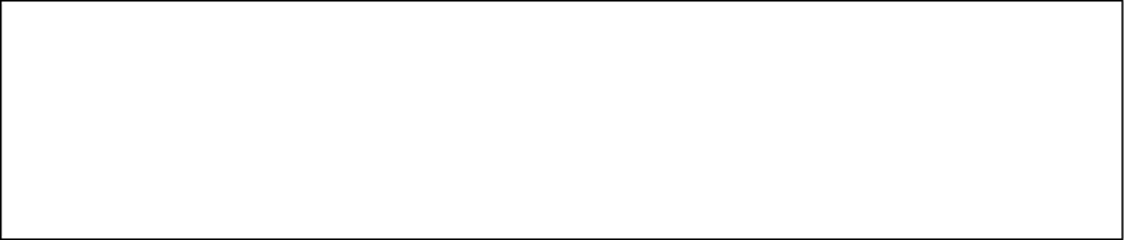


Date: ………………………………………………

Participants' Name and signature:  …………………………………………………………………….

Experimenter’s name and signature: …………………………………………………………………

This document is kept in the record by the project’s leader.

**APPENDIX 4 - Inclusion Criteria French**

Afin de minimiser les atteintes à la vie privée, nous énumérons tous les critères (d’inclusion et/ou d’exclusion) et vous demandons si vous les remplissez tous en même temps.

**Critères d’inclusion : Veuillez répondre OUI ou NON si vous remplissez ou non tous les points énumérés :**

o   Vous avez entre 18 et 60 ans,

o   Vous avez un niveau d’étude baccalauréat minimum,

o   Vous avez une vision et une audition normales (ou corrigées),

o   Vous êtes affilié à la sécurité sociale,

o   Vous avez signé un formulaire de consentement éclairé.

**Réponse : .................**

**Critères d’exclusion : Veuillez répondre OUI ou NON si vous remplissez ou non tous les points énumérés :**

- Personne protégée ;
- Présence d’un trouble neuropsychologique connu ;
- Déficience visuelle ou auditive importante ;
- Prise de médicaments ou de substances psychotropes ;
- Épilepsie ;
- Allaitement ;
- maux de têtes fréquents ;
- Mal des transports.
- daltonisme

**Réponse : .................**


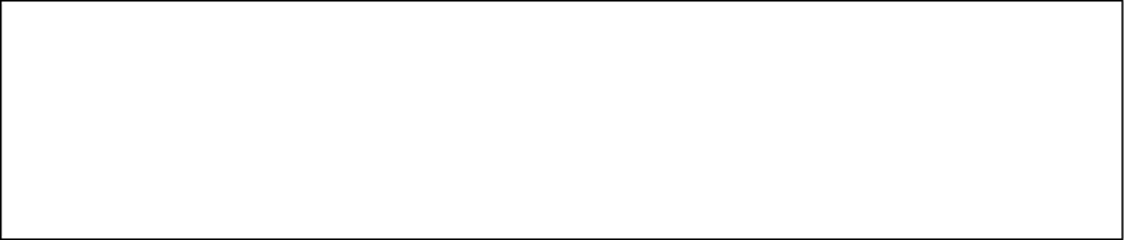


**Date :** ......................................................

**Nom et signature du participant :** ....................................................................................

**Nom et signature de l’expérimentateur :** .........................................................................

Ce document est conservé dans le dossier par le responsable du projet.

**APPENDIX 5  - Demographics Questionnaire**

**
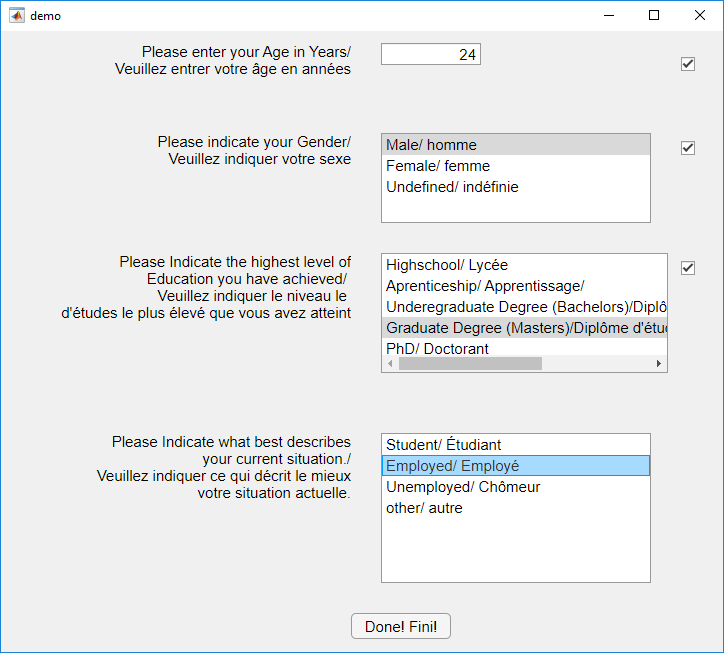
**

**APPENDIX 6 - SPS**

**
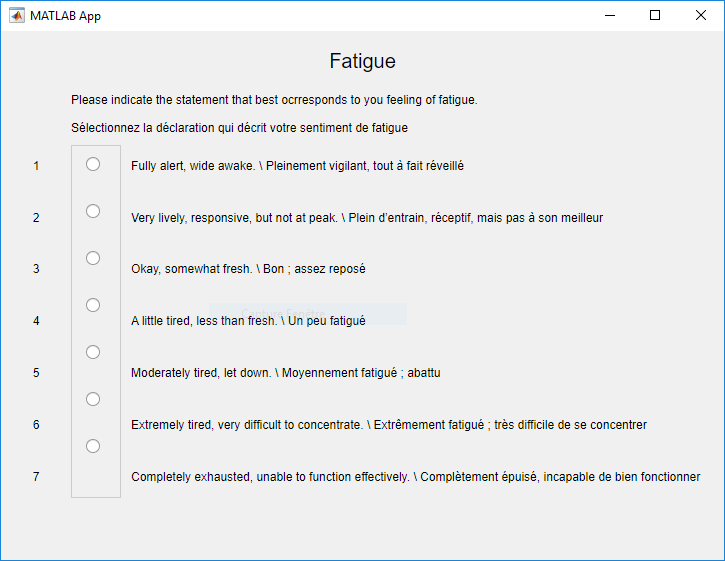
**

**APPENDIX 7 - KSS**


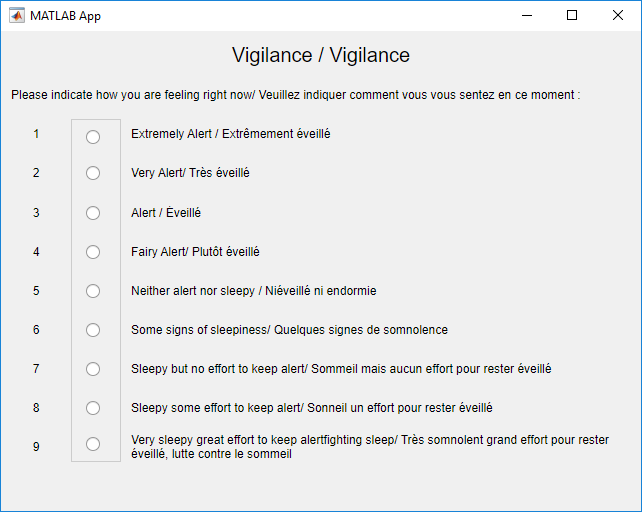


**APPENDIX 8 - Edinburgh Handedness Questionnaire**

# **
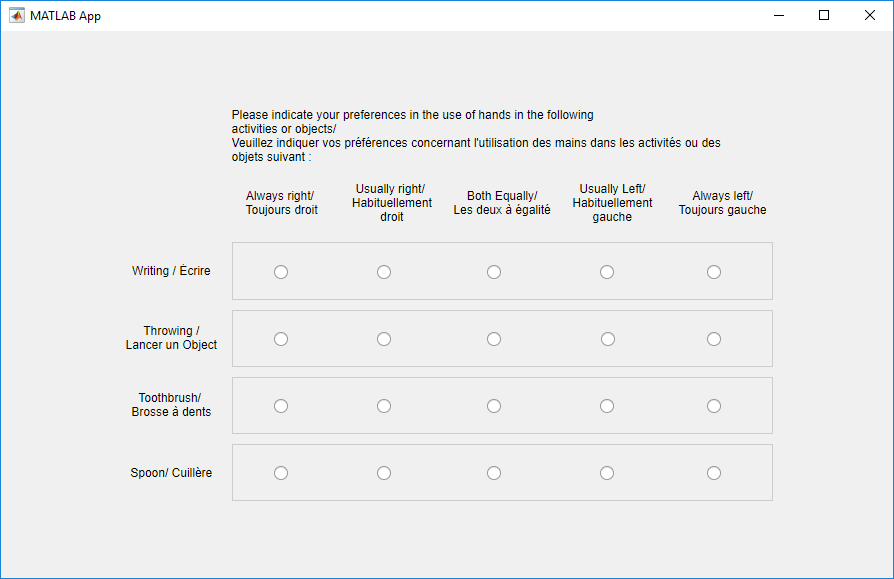
**

**APPENDIX 9 Ishihara Test**

NOTE: Only a few examples are presented, the complete test encompasses 38 plates.

chrome-extension://efaidnbmnnnibpcajpcglclefindmkaj/https://www.challengetb.org/publications/tools/country/Ishihara_Tests.pdf


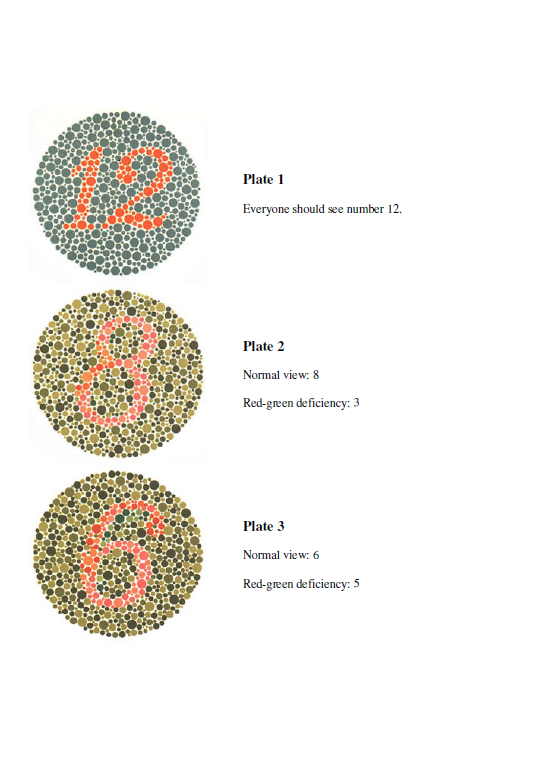


**APPENDIX 10 St. Mary’s Hospital Sleep Questionnaire**

 
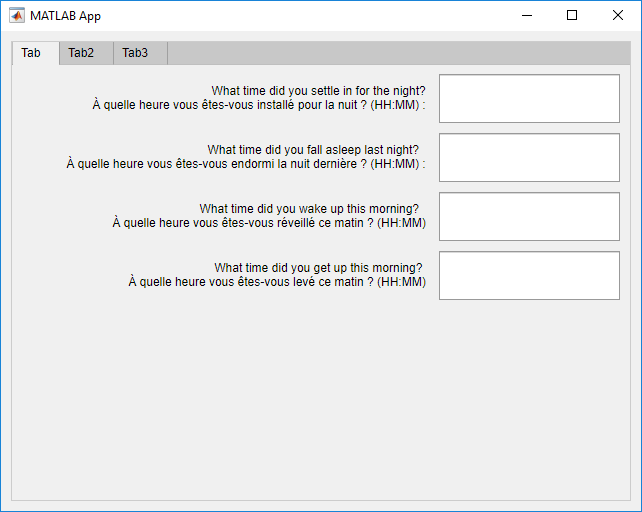


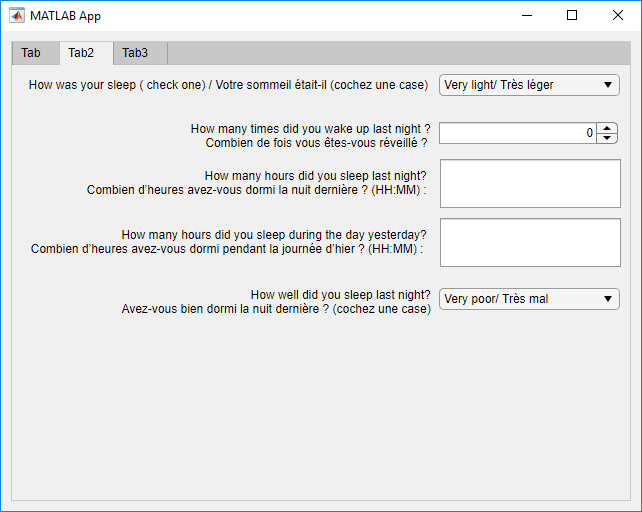


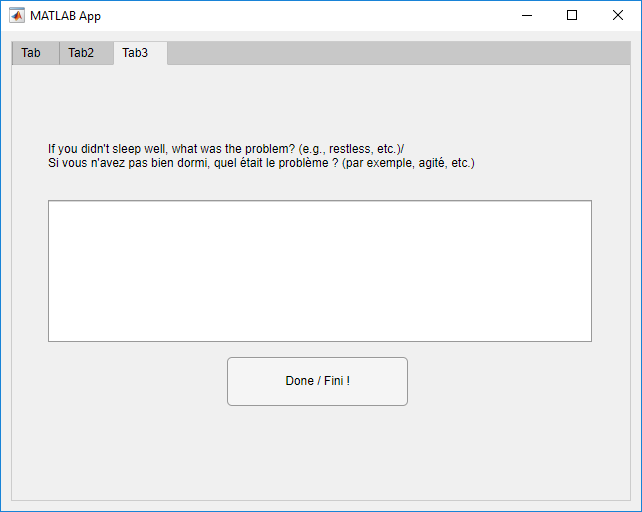


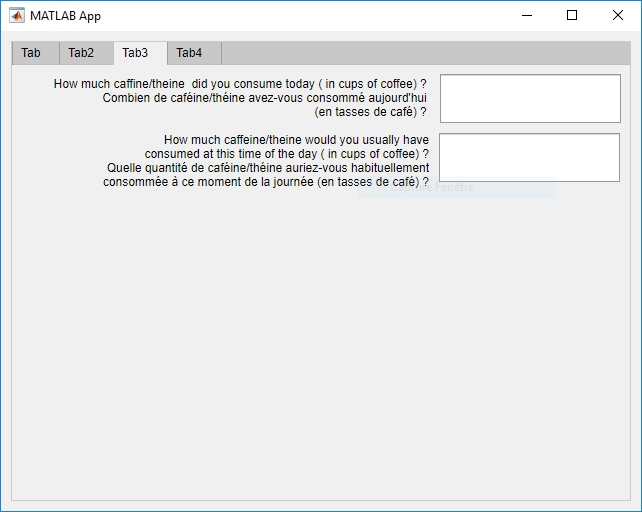


This questionnaire is about your sleep during the last 24 hours. Please try to answer each question./ Ce questionnaire porte sur votre sommeil au cours des dernières 24 heures. Veuillez essayer de répondre à chaque question.

·         What time did you settle in for the night/ À quelle heure vous êtes-vous installé pour la nuit ? (HH:MM) : _______

·       What time did you fall asleep last night?   À quelle heure vous êtes-vous endormi la nuit dernière ? (HH:MM) : _______

·        What time did you wake up this morning?   À quelle heure vous êtes-vous réveillé ce matin ? (HH:MM) : _______

·       What time did you get up this morning?  À quelle heure vous êtes-vous levé ce matin ? (HH:MM) : _______

How was your sleep ( check one) /Votre sommeil était-il (cochez une case) :

o   Very light/ Très léger

o   Light /Léger

oFairly light/   Assez léger

o   Moderately light /Léger moyen

o   Moderately deep/ Profond moyen

o   Fairly deep/Assez profond

o   Deep/ Profond

o   Very deep/ Très profond

How many times did you wake up last night (check one)? /Combien de fois vous êtes-vous réveillé ? (cochez une case)

- Not at all/ Pas du tout
- 1
- 2
- 3
- 4
- 5
- 6
- More than 6 times/  Plus de six fois

How many hours did you sleep last night? / Combien d’heures avez-vous dormi la nuit dernière ? (HH:MM) : _______

How many hours did you sleep during the day yesterday? /Combien d’heures avez-vous dormi pendant la journée d’hier ? (HH:MM) : _______

How well did you sleep last night?/ Avez-vous bien dormi la nuit dernière ? (cochez une case)

o  Very poor/ Très mal

o   Poor/ Mal

o   Fairly poor/ Assez mal

o  Fairly good/  Assez bien

o   Good/ Bien

o   Very good/ Très bien

How much caffine/theine did you consume today ( in cups of coffee) ? /Combien de caféine/théine avez-vous consommé aujourd'hui (en tasses de café) ?

How much caffeine/theine would you usually have consumed at this time of the day ( in cups of coffee) ? Quelle quantité de caféine/théine auriez-vous habituellement consommée à ce moment de la journée (en tasses de café) ?

If you didn't sleep well, what was the problem? (e.g., restless, etc.)/ Si vous n'avez pas bien dormi, quel était le problème ? (par exemple, agité, etc.)
